# Effect of α-Lipoic Acid on Lipid Profile in Rats Fed a High-Fructose Diet

**DOI:** 10.1080/15438600490486778

**Published:** 2004

**Authors:** V. Thirunavukkarasu, A. T. Anitha Nandhini, C. V. Anuradha

**Affiliations:** 1 Department of Biochemistry Faculty of Science Annamalai University Annamalai Nagar Tamil Nadu 608 002 India

## Abstract

This study investigated the effect of administration of *α*-lipoic acid (LA) on lipid metabolism in high fructose–fed insulin-resistant rats. High-fructose feeding (60 g/100 g diet) to normal rats resulted in a significant increase in the concentrations of cholesterol, triglycerides (TGs), free fatty acids (FFAs), and phospholipids in plasma, liver, kidney, and skeletal muscle. Reduced activities of lipoprotein lipase (LPL) and lecithin cholesterol acyl transferase (LCAT) and increased activity of the lipogenic enzyme hydroxymethylglutaryl–coenzyme A (HMG-CoA) reductase were observed in plasma and liver. High-density lipoprotein cholesterol (HDL-C) was significantly lowered and very low-density lipoprotein cholesterol (VLDL-C) and low-density lipoprotein cholesterol (LDL-C) were significantly elevated. Treatment with LA (35 mg/kg body weight intraperitoneal) reduced the effects of fructose. The rats showed near-normal levels of lipid components on plasma and tissues. Activities of key enzymes of lipid metabolism were also restored to normal values. Cholesterol distribution in the plasma lipoproteins was normalized, resulting in a favorable lipid profile. This study demonstrates that LA can alter lipid metabolism in fructose-fed insulin-resistant rats and may have implications in the treatment of insulin resistance.


*Experimental Diab. Res.*, 5:195–200, 2004
Copyright © Taylor and Francis Inc.
ISSN: 1543-8600 print / 1543-8619 online
DOI: 10.1080/15438600490486778

Taylor & Francis
Taylor & Francis Group

### Effect of $\alpha$-Lipoic Acid on Lipid Profile in Rats Fed a High-Fructose Diet

**V. Thirunavukkarasu, A. T. Anitha Nandhini, and C. V. Anuradha**

*Department of Biochemistry, Faculty of Science, Annamalai University, Annamalai Nagar, India*

**This study investigated the effect of administration of $\alpha$-lipoic acid (LA) on lipid metabolism in high fructose–fed insulin-resistant rats. High-fructose feeding (60 g/100 g diet) to normal rats resulted in a significant increase in the concentrations of cholesterol, triglycerides (TGs), free fatty acids (FFAs), and phospholipids in plasma, liver, kidney, and skeletal muscle. Reduced activities of lipoprotein lipase (LPL) and lecithin cholesterol acyl transferase (LCAT) and increased activity of the lipogenic enzyme hydroxymethylglutaryl–coenzyme A (HMG-CoA) reductase were observed in plasma and liver. High-density lipoprotein cholesterol (HDL-C) was significantly lowered and very low-density lipoprotein cholesterol (VLDL-C) and low-density lipoprotein cholesterol (LDL-C) were significantly elevated. Treatment with LA (35 mg/kg body weight intraperitoneal) reduced the effects of fructose. The rats showed near-normal levels of lipid components on plasma and tissues. Activities of key enzymes of lipid metabolism were also restored to normal values. Cholesterol distribution in the plasma lipoproteins was normalized, resulting in a favorable lipid profile. This study demonstrates that LA can alter lipid metabolism in fructose-fed insulin-resistant rats and may have implications in the treatment of insulin resistance.**

**Keywords** Cholesterol; Fructose; Insulin Resistance; $\alpha$-Lipoic Acid; Lipoproteins

Feeding rats with high dosage of fructose (>60% of total calories in diet) affects lipid metabolism and causes profound changes in plasma lipid profile. Fructose elevates triglycerides (TGs) and cholesterol in blood [1]. Fructose feeding evokes significant alterations particularly in liver TG metabolism and is reported to be atherogenic due to induction of lipogenic enzymes in liver [2]. Lipid changes associated with fructose feeding are documented to be secondary to the development of insulin resistance and loss of in vivo insulin sensitivity [3]. Loss of insulin action causes a shift in balance from oxidation to esterification of free fatty acids (FFAs), resulting in elevated very low-density lipoprotein (VLDL) secretion [4].

$\alpha$-Lipoic acid (LA) is a natural cofactor in dehydrogenase complexes such as pyruvate dehydrogenase and $\alpha$-ketoglutarate dehydrogenase. It is a small molecule that can easily cross the blood-brain barrier and has antioxidant properties [5]. LA improves insulin sensitivity and is found to be useful in the treatment of insulin resistance [6], LA has been prescribed for diabetic complications, especially diabetic polyneuropathy, in Germany for over 30 years [7]. LA influences lipid metabolism in liver, kidney, and blood of renal stone–forming rat model [8, 9]. However, there are no reports in the literature on the effects of LA on lipid parameters in the insulin-resistant state. Based on these observations, we designed a study to investigate the effect of LA on plasma and tissue lipid metabolism in fructose-fed insulin-resistant rats. The results obtained are compared with those in untreated fructose-fed rats.

### MATERIALS AND METHODS

### Animals and Diet

Male adult Wistar rats of body weight ranging from 150 to 170 g were obtained from the Department of Experimental Medicine, Central Animal House, Rajah Muthiah Medical

Received 21 January 2004; accepted 31 March 2004.
Address correspondence to Dr. C. V. Anuradha, Reader, Department of Biochemistry, Faculty of Science, Annamalai University, Annamalai Nagar, Tamil Nadu-608 002, India. E-mail: cvaradha@hotmail.com

College, Annamalai Nagar. Rats were housed 2 per cage under controlled conditions on a 12-hour light/12-hour dark cycle. Animals received a standard pellet diet (Karnataka State Agro Corporation, Agro Feeds Division, Bangalore, India) and water ad libitum. The animals used in the present study were treated under the principles and guidelines of the Ethical Committee of Animal Care of Annamalai University and in accordance with the Indian National Law on animal care and use.

After acclimatization the animals were divided into the following 4 groups consisting of 6 rats each.

| | |
|---|---|
| Group 1 (CON): | Received control diet and water ad libitum; 0.2 mL of saline was given intraperitoneally. |
| Group 2 (FRU): | Received a fructose-enriched diet and water ad libitum; 0.2 mL of saline was given intraperitoneally. |
| Group 3 (FRU + LA): | Received fructose diet and administered with lipoic acid (35 mg/kg body weight [bw]) in saline by intraperitoneal injection. |
| Group 4 (CON + LA): | Received the control diet and were given lipoic acid (35 mg/kg bw) in saline by intraperitoneal injection. |

The composition of control diet and fructose diet are given in Table 1. The diets were prepared fresh daily.

The animals were maintained in their respective groups for 20 days. At the end of 20 days, the rats were fasted overnight (12 hours) and were anesthetized using diethyl ether. Blood samples were collected by sinocular puncture in heparinized tubes and the animals were sacrificed by cervical dislocation. Plasma was separated by centrifugation. Liver, kidney, and skeletal muscle tissues were collected, washed in ice-cold saline, homogenized in 0.1 M Tris–hydrochloric acid buffer, pH 7.4, and used for various estimations.

**TABLE 1**
Composition of diet (g/100 g)

| Ingredients | Control diet | High-fructose diet |
|---|---|---|
| Corn starch | 60 | — |
| Fructose | — | 60 |
| Casein (fat free) | 20 | 20 |
| Methionine | 0.7 | 0.7 |
| Groundnut oil | 5 | 5 |
| Wheat bran | 10.6 | 10.6 |
| Salt mixture[a] | 3.5 | 3.5 |
| Vitamin mixture[b] | 0.2 | 0.2 |

[a]The composition of mineral mix (g/kg): $MgSO_4{\cdot}7H_2O$ 30.5; NaCl 65.2; KCl 105.7; $KH_2PO_4$ 200.2; $3MgCO_3$, $Mg(OH)_2{\cdot}3H_2O$ 38.8; $FeC_6H_5O_7{\cdot}5H_2O$ 40.0; $CaCO_3$ 512.4; KI 0.8; NaF 0.9; $CuSO_4.5H_2O$ 1.4; $MnSO_4$ 0.4, and $CONH_3$ 0.05.

[b]One kilogram of vitamin mix contained thiamine mono nitrate, 3 g; riboflavin, 3 g; pyridoxine HCI, 3.5 g; nicotinamide, 15 g; *d*-calcium pantothenate, 8 g; folic acid, 1 g; *d*-biotin, 0.1 g; cyanocobalamin, 5 mg; vitamin A acetate, 0.6 g; $\alpha$-tocopherol acetate, 25 g; and choline chloride, 10 g.

### Lipid Analysis

Lipids in plasma and tissues were extracted by the method of Folch and colleagues [10]. Total lipids were extracted with chloroform methanol mixture 2:1 (*v*/*v*) after homogenizing the tissue. Aliquots of the lipid extracts were evaporated to dryness and used for the estimation of lipids. Estimation of cholesterol, TGs, phospholipids, FFAs, and concentration of cholesterol in plasma lipoproteins were carried out following the procedures described earlier [11].

Lipoprotein lipase (LPL) was assayed in plasma by the method of Korn [12]. Lecithin cholesterol acyl transferase (LCAT) was assayed in plasma by the method of Hitz and colleagues [13]. Hydroxymethylglutaryl–coenzyme A (HMG-CoA) reductase activity was measured by the method of Rao and Ramakrishnan [14].

### Statistical Analysis

Values are expressed as means ± SD. Data was analysed using one-way analysis of variance (ANOVA) followed by Duncan's multiple range test (DMRT). A value of $P < .05$ was considered statistically significant.

### RESULTS

The plasma lipid concentrations were found to be significantly increased in rats fed high fructose (Figure 1). Rats simultaneously treated with LA along with fructose (FRU+LA) showed significant reductions as compared to fructose-treated rats.

Concentrations of lipids in liver, kidney, and skeletal muscle of control and experimental animals are given in Figures 2, 3, and 4, respectively. TG, cholesterol, and FFA concentrations were significantly increased in fructose-fed rats as compared to normal rats. The phospholipid concentrations were significantly decreased in fructose-fed rats. LA administration brought the concentrations of lipid constituents in tissues to near normal in fructose-treated rats.

Table 2 summarizes the distribution of cholesterol in plasma lipoprotein fractions. The concentrations of VLDL-cholesterol (VLDL-C) and low-density lipoprotein LDL-cholesterol (LDL-C) were significantly increased whereas high-density lipoprotein cholesterol (HDL-C) was lowered in fructose-fed rats.

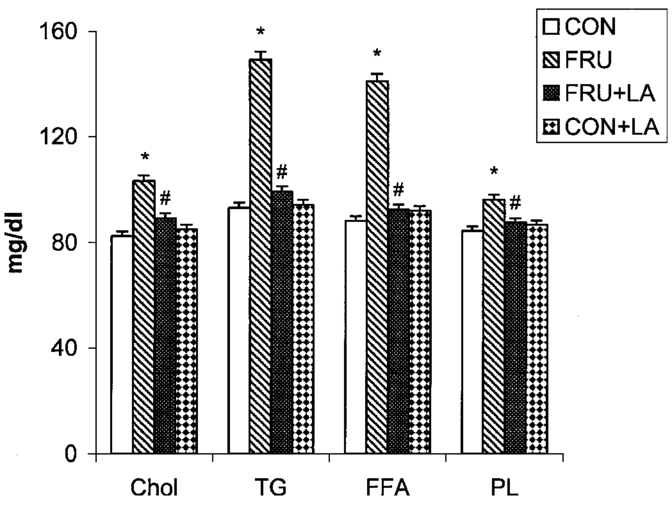

**FIGURE 1**
Concentration of cholesterol, TG, FFA, and phospholipids in plasma of the experimental animals. Values are means ± SD (n = 6 in each group). *$P < .05$ as compared to control; #$P < .05$ as compared to fructose. CON: control; FRU: fructose; LA: lipoic acid; Chol: cholesterol; TG: triglycerides; FFA: free fatty acids; PL: phospholipids.

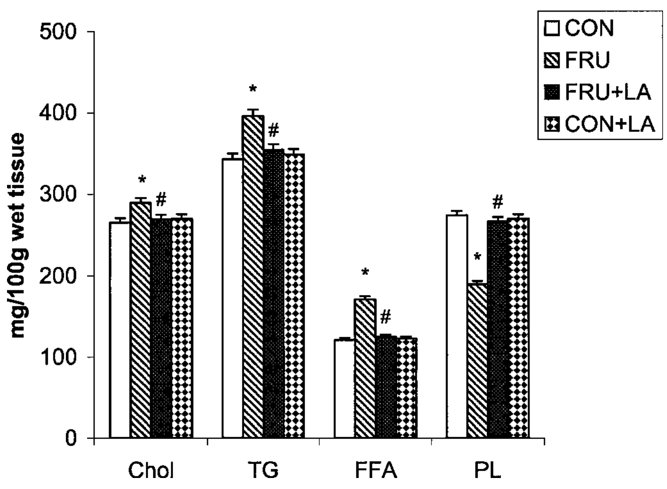

**FIGURE 3**
Concentration of cholesterol, TG, FFA, and phospholipids in kidney of the experimental animals. Values are means ± SD (n = 6 in each group). *$P < .05$ as compared to control; #$P < .05$ as compared to fructose. CON: control; FRU: fructose; LA: lipoic acid; Chol: cholesterol; TG: triglycerides; FFA: free fatty acids; PL: phospholipids.

These alterations were reversed and the cholesterol distribution in lipoprotein fractions in LA-treated rats was similar to that of control rats.

The activities of LPL, LCAT in plasma, and HMG-CoA reductase in liver are presented in the Table 3. The activities of LPL and LCAT were lowered in plasma and liver of fructose-fed rats. There was an increased activity of HMG-CoA reductase in fructose-fed group. The activities of these enzymes were restored to baseline control values when rats were treated with LA.

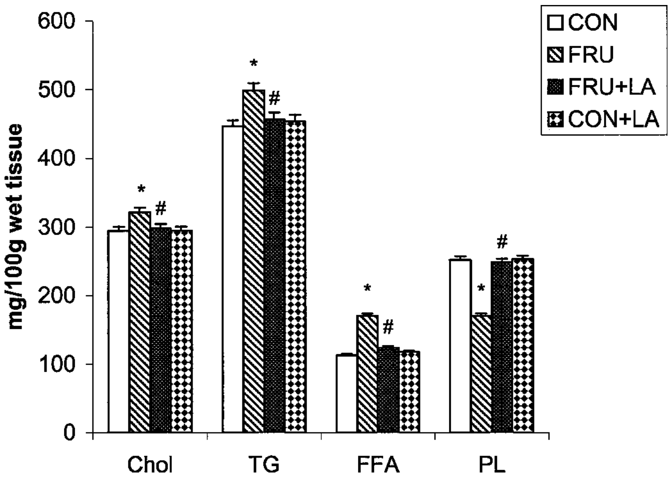

**FIGURE 2**
Concentration of cholesterol, TG, FFA, and phospholipids in liver of the experimental animals. Values are means ± SD (n = 6 in each group). *$P < .05$ as compared to control; #$P < .05$ as compared to fructose. CON: control; FRU: fructose; LA: lipoic acid; Chol: cholesterol; TG: triglycerides; FFA: free fatty acids; PL: phospholipids.

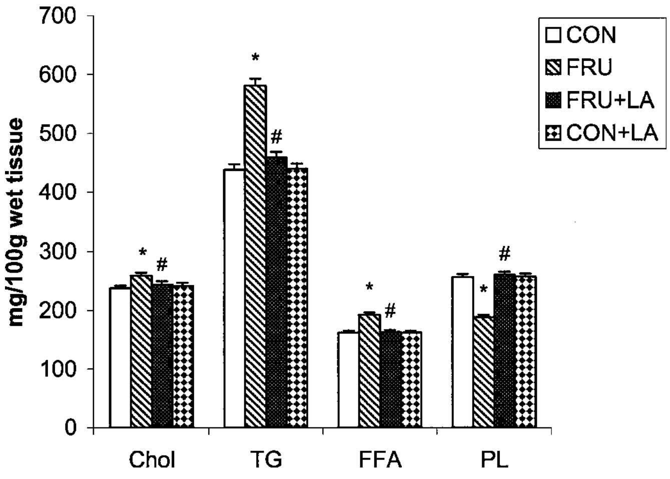

**FIGURE 4**
Concentration of cholesterol, TG, FFA, and phospholipids in muscle of the experimental animals. Values are means ± SD (n = 6 in each group). *$P < .05$ as compared to control; #$P < .05$ as compared to fructose. CON: control; FRU: fructose; LA: lipoic acid; Chol: cholesterol; TG: triglycerides; FFA: free fatty acids; PL: phospholipids.

**TABLE 2**
Distribution of cholesterol in the lipoprotein fractions (mg/dL)

| | CON | FRU | FRU + LA | CON + LA |
|---|---|---|---|---|
| HDL-cholesterol | 34.8 ± 2.8 | 28.8 ± 1.5[a] | 31.3 ± 0.8[b] | 35.0 ± 1.8 |
| LDL-cholesterol | 29.0 ± 6.0 | 44.8 ± 11.5[a] | 38.2 ± 6.2[b] | 31.2 ± 6.2 |
| VLDL-cholesterol | 18.6 ± 0.8 | 29.8 ± 1.5[a] | 19.8 ± 0.7[b] | 18.8 ± 0.7 |

*Note*. Values are means ± SD of 6 rats from each group. CON = control rats; FRU = fructose-fed rats; FRU + LA = fructose-fed rats treated with lipoic acid; CON + LA = control rats treated with lipoic acid.
[a]Significant as compared with control rats ($P < .05$) (DMRT).
[b]Significant as compared with fructose-fed rats ($P < .05$) (DMRT).

LA administration to normal rats did not produce significant alterations in the enzyme activities or lipid components.

### DISCUSSION

Lipid changes observed in fructose-treated rats were elevated levels of cholesterol, TGs, FFAs, and phospholipids in plasma. HDL-C was decreased whereas there was a significant increase in LDL-C and VLDL-C. Accumulation of cholestrol, TGs, and FFAs, was observed in tissues. These findings are consistent with our previous reports [3, 11] and with those of other investigators [15, 16]. Induction of lipogenic enzyme HMG-CoA reductase and alterations in lipid transport enzymes are also noticed in the present study. Feeding a high-fructose diet to diabetic rats produces an increase in activity of HMG-CoA reductase and addition of fructose to cultured rat hepatocytes increases HMG-CoA reductase by approximately 3-fold [17].

We have previously reported that fructose feeding induces hyperinsulinemia and insulin resistance [18]. The elevated TG concentrations can be associated with impaired insulin action. Bieger and colleagues [19] have shown the increase in blood TG concentrations can decrease insulin sensitivity by reducing the number of insulin receptors. A causative link between elevated circulating TGs and impaired insulin action was observed in fructose-fed rats by Thorburn and colleagues [16].

Increased TG levels in fructose-fed rats might be attributed both to the overproduction of hepatic VLDL-TG and impaired peripheral clearance. Increased hepatic TG production might result from the conversion of fructose carbon into glycerol-3-phosphate, a precursor of lipid synthesis [2] or from increased esterification of circulatory nonesterified fatty acids. Several authors have reported increased hepatic lipogenesis as a consequence of dietary fructose [20, 21].

FFAs serve as important substrates for hepatic TG synthesis and high concentrations could lead to hypertriglyceridemia. Fiebig and colleagues [22] evidenced that high-fructose feeding may increase the availability of long-chain fatty acids required for synthesis of TGs by up-regulating the hepatic lipogenic enzymes, including hepatic fatty acid synthase and acetyl-CoA carboxylase. Furthermore, the acute inhibitory effect of insulin on hepatic VLDL secretion is modified by the ambient plasma FFA concentration. Significant reduction in the activity of LPL could be another reason for hypertriglyceridemia. Acute

**TABLE 3**
Activities of lipoprotein lipase (LPL) and lecithin cholesterol acyl transferase (LCAT) in plasma and hydroxymethylglutaryl (HMG)-CoA reductase in liver

| | CON | FRU | FRU + LA | CON + LA |
|---|---|---|---|---|
| LCAT ($\mu$moles of cholesterol/hr/L) | 75.3 ± 2.2 | 64.1 ± 2.3[a] | 72.8 ± 1.8[b] | 74.2 ± 1.2 |
| LPL ($\mu$moles of glycerol liberated/hr/L) | 5.7 ± 0.6 | 4.8 ± 0.5[a] | 5.3 ± 0.2[b] | 5.8 ± 0.7 |
| HMG-CoA reductase (Ratio of HMG-CoA to mevalonate)[c] | 3.1 ± 0.13 | 2.1 ± 0.2[a] | 3.1 ± 0.1[b] | 3.1 ± 0.1 |

*Note*. Values are means ± SD of 6 rats from each group. CON = control rats; FRU = fructose-fed rats; FRU + LA = fructose-fed rats treated with lipoic acid; CON + LA = control rats treated with lipoic acid.
[a]Significant as compared with control rats ($P < .05$) (DMRT).
[b]Significant as compared with fructose-fed rats ($P < .05$) (DMRT).
[c]Lower ratio indicates higher enzyme activity and vice versa.

administration of glucose elevates adipose tissue LPL activity whereas fructose fails to increase adipose tissue LPL activity [23].

The lowered HDL-C concentration in fructose-fed rats can be attributed to the decreased LPL and LCAT activities in plasma. LCAT, the enzyme that catalyzes esterification of cholesterol with FFAs, along with LPL is responsible for HDL-C synthesis. It plays an important role in cholesterol and TG transport and metabolism. The decreased activity of LCAT indicates impairment in HDL-C synthesis as well as TG metabolism in fructose-fed rats. The effect of fructose feeding on LCAT, LPL, and HMG-CoA reductase produces changes in lipid components, mainly in the concentrations of cholesterol, TGs, HDL-C, and VLDL-C. Hallfrisch and colleagues [24] demonstrated higher plasma cholesterol and LDL-C concentration in healthy and hyperinsulinemic subjects after 4 weeks of high-fructose diet.

The reduction in the activity of LPL, an insulin-sensitive enzyme, in fructose-fed rats can be ascribed to the insulin resistance induced by fructose. Fructose feeding can lead to a decrease in the ability of insulin to stimulate the activity of LPL [25]. Hepatic lipase, another key enzyme of lipid metabolism subject to regulation by insulin, is abundant in subluminal extracellular matrix of liver and hydrolyses TGs and phospholipids from lipoprotein. It could be possible that the activity of hepatic lipase is blocked in these rats due to hepatic insulin resistance.

High plasma FFA concentration impairs the action of insulin on glucose disposal via substrate competition in the glucose-FFA cycle. Conversely, diminished insulin-stimulated glucose disposal could lead to impaired FFA reesterification and thereby to higher circulating FFA concentrations. Elevated concentrations of plasma FFAs may play a key role in the pathogenesis of type 2 diabetes by impairing peripheral glucose utilization and by promoting hepatic glucose overproduction [26]. These data suggest that the action of insulin on FFA metabolism is impaired in hyperinsulinemia/insulin resistance state. This could explain higher TG concentrations in fructose-fed rats in the present study.

Fructose facilitates oxidative damage in tissues [27]. Increased plasma phospholipids and their depletion in tissues of fructose-fed rats may be attributed to oxidative stress. The major targets of damaging free radicals are the cellular and membrane phospholipids [28]. The oxidative tissue damage can release the membrane lipids such as FFAs and phospholipids into blood [29].

Supplementation of LA to rats fed high-fructose diet mitigated these alterations. The levels of plasma and tissue lipids were lowered significantly when LA was administered along with fructose. HDL-C concentration was increased whereas those of LDL-C and VLDL-C were decreased.

These effects of LA on lipid metabolism may be related to its effect on glucose utilisation. LA increases glucose disposal, insulin sensitivity, and insulin action in fructose-treated rats [30]. This could result in efficient regulation of the key enzymes of lipid metabolism measured in the present study by normalizing the circulatory lipid concentrations. Lowering of plasma TG concentrations may be attributed to the reduced availability of the precursor FFAs and to enhanced peripheral tissue clearance through increased LPL activity. The rise in plasma concentrations of HDL-C in LA-treated fructose rats may be due to delayed clearance and increased synthesis of HDL constituents. Moreover stimulation of LPL leads to a rise in HDL production and reduction in VLDL constituents [31]. Maintenance of ambient levels of phospholipids in plasma and tissues of LA-treated fructose rats could be due to antioxidant property exerted by LA. LA quenches reactive oxygen species that could act on membranes of the biological systems [5].

LA is reported to favorably influence the plasma and tissue concentrations of lipids during Adriamycin-induced hyperlipidemia in rats [8]. LA has also marked effects in lowering vascular hemostatic and lipid risk factors in the streptozotocin-diabetic rats [32].

The results of the present study suggest that LA has modulatory effects on lipid metabolism in fructose-fed insulin-resistant rats. Risk factors for cardiovascular disease, such as dyslipidemia, hypertension, and glucose intolerance, tend to cluster within individuals and insulin resistance is a common feature in these conditions. A parallel between this nutritional-experimental model and the multimetabolic syndrome called syndrome X has been postulated by Reaven [33]. The present findings show that LA may have implications for the treatment of dyslipidemia associated with insulin resistance.

### REFERENCES

[1] Park, O. J., Cesar, D., Faix, D., Wu, K., Shackleton, C. H. L., and Hellerstein, M. K. (1992) Mechanism of fructose induced hypertriglyceridemia in rats. *Biochem. J.,* **282,** 753–757.

[2] Zavaroni, I., Sander, S., Scott, S., and Reaven, G. M. (1980) Effect of fructose feeding on insulin secretion and insulin action in the rat. *Metabolism,* **10,** 970–973.

[3] Srividhya, S., and Anuradha, C. V. (2002) Metformin improves liver antioxidant potential in rats fed a high-fructose diet. *Asia Pacific J. Clin. Nutr.,* **11,** 319–322.

[4] Mayes, P. A. (1993) Intermediary metabolism of fructose. *Am. J. Clin. Nutr.,* **58 (Suppl),** 754S–765S.

[5] Packer, L., Witt, K. H., and Tritschler, H. J. (1995) $\alpha$-Lipoic acid as a biological antioxidant. *Free Radic. Biol. Med.,* **19,** 227–250.

[6] Evans, J. L., and Goldfine, I. D. (2000) $\alpha$-Lipoic acid a multifunctional antioxidant that improves insulin sensitivity in patients with type 2 diabetes. *Diabetes Tech. Ther.,* **2,** 401–413.

[7] Ziegler, D., Reljanovic, M., Mehnert, H., and Gries, F. A. (1999) Alpha lipoic acid in the treatment of diabetic polyneuropathy in Germany: Current evidence from clinical trials. *Exp. Clin. Endocrinol. Diabetes,* **107,** 421–430.

[8] Malarkodi, K. P., Balachandar, A. V., and Varalakshmi, P. (2003) The influence of lipoic acid on adriamycin-induced hyperlipidemic nephrotoxicity in rats. *Mol. Cell. Biochem.,* **247,** 139–145.

[9] Sumathi, R., Jayanthi, S., and Varalakshmi, P. (1995) Impaired lipid metabolism in calcium oxalate stone forming rats and dl-$\alpha$-lipoic acid supplementation. *Nutr. Res.,* **15,** 59–70.

[10] Folch, J., Loer, M., and Stanley, G. S. H. (1951) A simple method for the isolation and purification of total lipids from animal tissue. *J. Biol. Chem.,* **126,** 497–509.

[11] Nandhini, A. T. A., Balakrishnan, S. D., and Anuradha, C. V. (2001) Taurine improves lipid profile in rats fed a high-fructose diet. *Nutr. Res.,* **22,** 343–348.

[12] Korn, E. D. (1955) Clearing factors: A heparin activated lipoprotein lipase. Isolation and characterization of enzyme from normal rats. *J. Biol. Chem.,* **215,** 1–26.

[13] Hitz, J., Sterinmetz, J., and Surt, G. (1983) Plasma LCAT-reference values and effects of xenobiotics. *Clin. Chim. Acta,* **133,** 85–96.

[14] Rao, V., and Ramakrishnan, S. (1975) Indirect assessment of hydroxy methyl glutaryl CoA reductase (NADH) activity in liver tissue. *Clin. Chem.,* **21,** 1523–1525.

[15] Michaelis, D. C., Nace, C. S., and Szepsi, B. (1975) Demonstration of a specific metabolic effect of dietary dissacharides in the rat. *J. Nutr.,* **105,** 1186–1191.

[16] Thorburn, A. W., Storlein, L. H., Jenkins, A. B., Khouri, S., and Kraegen, E. W. (1989) Fructose induced in vitro insulin resistance and elevated plasma triglyceride levels in rats. *Am. J. Clin. Nutr.,* **49,** 1155–1163.

[17] Spence, J. T., Koudelka, A. P., and Tseng-Crank J. C. (1985) Role of protein synthesis in the carbohydrate-induced changes in the activities of acetyl CoA-carboxylase and hydroxymethylglutaryl CoA reductase in cultured rat hepatocytes. *Biochem. J.,* **227,** 939–947.

[18] Anuradha, C. V., and Balakrishnan, S. D. (1999) Taurine attenuates hypertension and improves insulin sensitivity in the fructose-fed rats, an animal model of insulin resistance. *Can. J. Physiol. Pharmacol.,* **77,** 749–754.

[19] Bieger, W. P., Michel, G., Barwich, D., and Wirth, A. (1984) Diminished insulin receptors on monocytes and erythrocytes in hypertriglyceridemia. *Metabolism,* **33,** 982–987.

[20] Boogaerts, J. R., Malone-McNeal, M., Archambault Schexnayder, J., and Davis, P. A. (1984) Dietary carbohydrates induces lipogenesis and very low density lipoproteins synthesis. *Am. J. Physiol.,* **246,** E77–E83.

[21] Carmona, A., and Freedland, R. A. (1989) Comparison among the lipogenic potential of various substrates in rat hepatocytes: The differential effects of fructose-containing diets on hepatic lipogenesis. *J. Nutr.,* **119,** 1304–1310.

[22] Fiebig, R., Griffiths, M. A., Gore, M. T., Baker, D. H., Oscai, L., Ney, D. M., and Ji, L. L. (1998) Exercise training down-regulates hepatic lipogenic enzymes in meal-fed rats: Fructose versus complex-carbohydrate diets. *J. Nutr.,* **128,** 810–817.

[23] Bar On, H., and Stein, Y. (1967) Effect of glucose and fructose administration on lipid metabolism in the rat. *J. Nutr.,* **94,** 95–105.

[24] Hallfrisch, J., Reiser, S., and Prather, E. S. (1983) Blood lipid distribution of hyperinsulinemic men consuming three levels of fructose. *Am. J. Clin. Nutr.,* **37,** 740–748.

[25] Anurag, P., and Anuradha, C. V. (2000) Metformin improved lipid metabolism and attenuates lipid peroxidation in high fructose-fed rats. *Diabetes Obes. Metab.,* **4,** 36–42.

[26] Boden, G., Jadalin, F., and Kian, Y. (1991) Effect of fat on insulin stimulated carbohydrate metabolism in normal men. *J. Clin. Invest.,* **88,** 960–966.

[27] Thirunavukkarasu, V., Nandhini, A. T., and Anuradha, C. V. (2003) Lipoic acid restores antioxidant system in tissues of hyperinsulinemic rats. *Indian J. Med. Res.,* **118,** 134–140.

[28] Slatter, D. A., Bolton, C. N., and Bailey, A. J. (2000) The importance of lipid-derived malondialdehyde in diabetes mellitus. *Diabetologia,* **43,** 550–557.

[29] Ohara, Y., Peterson, T. E., and Harrison, D. G. (1993) Hypercholesterolemia increases endothelial superoxide anion production. *J. Clin. Invest.,* **91,** 2546–2551.

[30] Vasdev, S., Fora, C. A., Parai, S., Longerich, L., and Gadag, V. (2000) Dietary lipoic acid supplementation prevents fructose-induced hypertension in rats. *Nutr. Metab. Cardiovasc. Dis.,* **10,** 339–346.

[31] Mochizuki, K., Oda, H., and Yokogoshi, H. (1998) Increasing effect of dietary taurine on the serum HDL-cholesterol concentration in rats. *Biosci. Biotech. Biochem.,* **62,** 578–579.

[32] Ford, I., Cotter, M. A., Greaves, M., and Cameron, N. E. (2001) The effects of treatment with alpha-lipoic acid or evening primrose oil on vascular hemostatic and lipid risk factors, blood flow, and peripheral nerve conduction in the streptozotocin-diabetic rat. *Metabolism,* **50,** 868–875.

[33] Reaven, G. M. (1991) Insulin resistance, hyperinsulinemia, hypertriglyceridemia and hypertension: Parallels between human diseases and animal models. *Diabetes Care,* **14,** 195–202.

